# Supplementary material for: A Comprehensive Assessment of Ultraviolet-Radiation-Induced Mutations in Flammulina filiformis Using Whole-Genome Resequencing
Source: J Fungi (Basel). 2024 Mar 20;10(3):228. doi: 10.3390/jof10030228 (PMC10971301; doi:10.3390/jof10030228)
Supplement: Supplementary file 1 [file jof-10-00228-s001.zip › Supplementary Material S8/KEGG annotation/out/64381550635650.os/KO/out_map/map04976.html]

KEGG PATHWAY: Bile secretion - Reference pathway


|  |  |
| --- | --- |
| **Bile secretion - Reference pathway** |  |

[
Pathway menu
| Organism menu
| Pathway entry
| Show description
| User data mapping
]

Bile is a vital secretion, essential for digestion and absorption of fats and fat-soluble vitamins in the small intestine. Moreover, bile is an important route of elimination for excess cholesterol and many waste product, bilirubin, drugs and toxic compounds. Bile secretion depends on the function of membrane transport systems in hepatocytes and cholangiocytes and on the structural and functional integrity of the biliary tree. The hepatocytes generate the so-called primary bile in their canaliculi. Cholangiocytes modify the canalicular bile by secretory and reabsorptive processes as bile passes through the bile ducts. The main solutes in bile are bile acids, which stimulate bile secretion osmotically, as well as facilitate the intestinal absorption of dietary lipids by their detergent properties. Bile acids are also important signalling molecules. Through the activation of nuclear receptors, they regulate their own synthesis and transport rates.

|  |  |
| --- | --- |
| Reference pathway | 100% |
